# Aspiration prevention surgeries: a review

**DOI:** 10.1186/s12931-023-02354-0

**Published:** 2023-02-06

**Authors:** Rumi Ueha, Redentor B. Magdayao, Misaki Koyama, Taku Sato, Takao Goto, Tatsuya Yamasoba

**Affiliations:** 1 Swallowing Center, The University of Tokyo Hospital, 7-3-1 Hongo, Bunkyo-Ku, Tokyo, 113-8655 Japan; 2 Department of Otolaryngology and Head and Neck Surgery, Faculty of Medicine, The University of Tokyo, Tokyo, Japan; 3 Department of Otorhinolaryngology-Head and Neck Surgery, Eastern Visayas Medical Center, Tacloban, Philippines

**Keywords:** Aspiration prevention surgery, Aspiration pneumonia, Surgery, Dysphagia, Swallowing

## Abstract

**Background:**

Severe dysphagia can cause intractable pneumonia and lead to life-threatening conditions. Intractable aspiration can occur despite medical management for aspiration prevention. Surgical intervention is indicated for intractable aspiration to prevent potentially life-threatening complications. Since the 1970s, several surgical treatments to prevent aspiration have been reported, and various aspiration prevention surgeries have been introduced, but little is known about them or their benefits. This is a review of the types of aspiration prevention surgery, with the aim of increasing aspiration prevention surgery awareness and their clinical outcomes among medical professionals, which will guide the choices of aspiration prevention surgeries for patients with intractable aspiration.

**Main body:**

Aspiration prevention surgeries can be categorized into three according to their approaches: removal of the larynx, altering the structure of the trachea, and closure of the larynx. Aspiration prevention surgeries to remove the larynx include total and central-part laryngectomy. Aspiration prevention surgeries to alter the structure of the trachea include tracheoesophageal diversion, laryngotracheal separation, and the tracheal flap method. Surgeries to close the larynx can be divided into supraglottic laryngeal closure, glottic laryngeal closure, and subglottic laryngeal closure. Aspiration prevention surgeries prevent aspiration and increase oral intake in 50–80% of patients. Most patients lose vocal function after aspiration prevention surgeries; however, some patients who have undergone total laryngectomy or laryngotracheal separation restored their speech function through tracheoesophageal puncture and use of voice prosthesis. Postoperative suture failure is frequent after epiglottic flap closure and total laryngectomy but rare after central-part laryngectomy, laryngotracheal separation, glottic closure, and subglottic closure. Furthermore, aspiration prevention surgeries improve the quality of life of patients and their caregivers by decreasing suctioning frequency.

**Conclusions:**

In this review, we described the history and development of aspiration prevention surgeries. Medical professionals need to continually improve their knowledge and skills to facilitate appropriate aspiration prevention surgeries according to patient condition.

## Introduction

The larynx plays important roles in various body functions, which include respiration, speech, and protection of the lower airway. Impaired laryngeal function is associated with dysphagia and intratracheal aspiration (aspiration). Dysphagia is associated with aging and neuromuscular, cerebrovascular, esophageal, and laryngopharyngeal disorders. Aspiration can occur when there is laryngeal penetration of secretions such as saliva, ingested liquids or solids, or refluxed gastric contents below the level of the true vocal folds [1–3]. Severe dysphagia can cause intractable pneumonia and lead to life-threatening conditions. Individuals with decreased airway sensation are vulnerable to asymptomatic aspiration because the airway protective response against aspiration is impaired, resulting in poor airway clearance and the above-mentioned complications [3, 4].

Intractable aspiration occurs even when medical strategies, such as thickening liquids and modifying the texture of foods, are used to prevent aspiration [4–6]. Surgical intervention is indicated for intractable aspiration to prevent potentially life-threatening complications. Since the 1970s, several surgical treatments to prevent aspiration have been reported, and to date, various aspiration prevention surgeries (APSs) have been introduced [3–8], but only a few review articles have summarized APSs since 2010.

The purpose of this paper was to review the types of APSs and disseminate more detailed information about them and their clinical outcomes to more medical professionals. The insights from this study will guide medical professionals in choosing APSs for their patients with intractable aspiration.

## Surgical management for dysphagia

Surgical procedures for patients with severe dysphagia can be categorized into three according to their purpose: tracheostomy to create a route for suctioning aspirated material and secretions from the lower respiratory tract through the trachea; swallowing function improvement surgeries to improve pharyngeal swallowing while preserving speech function; APS to prevent aspiration despite loss of speech function.

### Tracheostomy

Tracheostomy is a common initial surgical approach to secure the airway and reduce aspiration. It is a useful adjunct for facilitating pulmonary toilets and reducing pulmonary dead space [5]. However, a cuffed tracheostomy tube alone is not reliable for preventing aspiration. A tracheostomy tube impairs laryngeal elevation and the production of an effective cough [5]. A large inflated tracheostomy cuff can transmit pressure to the cervical esophagus, which causes physiological obstruction [6]. Swallowing may also be impaired by a tracheostomy due to a decrease in pharyngeal pressures secondary to a leak through the tracheostomy opening. Lastly, chronic bypass of the upper airway with the tracheostomy can impair laryngeal closure reflex [5].

### Swallowing function improvement surgeries

The typical procedures under this category include pharyngeal flap surgery for velopharyngeal insufficiency, cricopharyngeal myotomy for cricopharyngeal dysfunction and upper esophageal sphincter disorders [9, 10], and laryngeal suspension for impaired laryngeal elevation [8, 11]. Various combinations of procedures can be performed depending on the pathophysiology [12, 13]. Needless to say, postoperative rehabilitation is indispensable after any surgery to improve swallowing function, as practicing swallowing safely with the new pharyngeal structure is necessary.

### Aspiration prevention surgeries

**Fig. 1:**
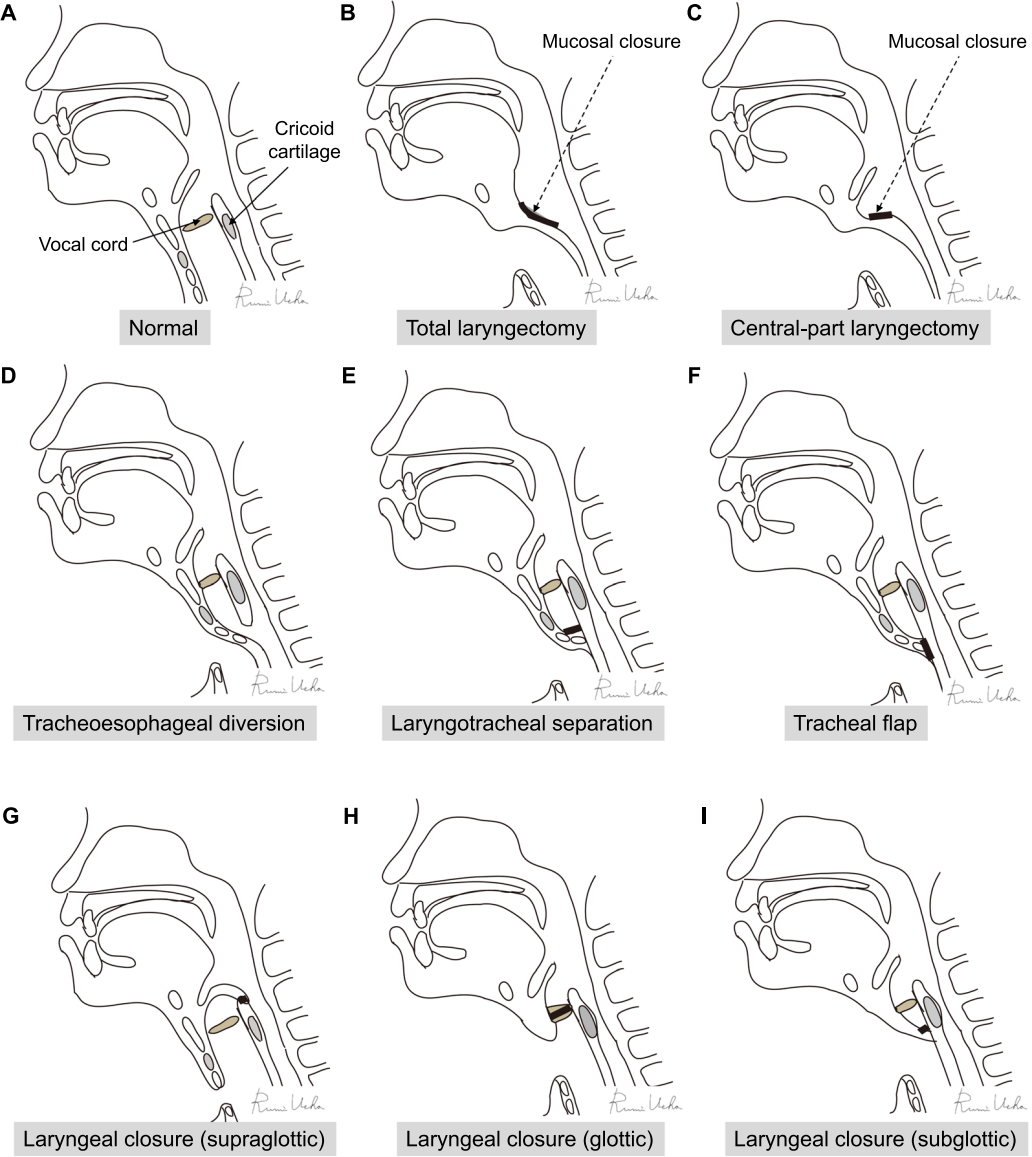
Schemas for various aspiration prevention surgeries. **A** normal, **B** total laryngectomy, **C** central-part laryngectomy, **D** tracheoesophageal diversion, **E** laryngotracheal separation, **F** tracheal flap method, **G** supraglottic laryngeal closure, **H** glottic laryngeal closure, **I** subglottic laryngeal closure

APSs are performed to change the pharyngolaryngeal structure for aspiration prevention [3–8]. There are several types of APSs, such as removal of the larynx, altering the structure of the trachea, and closure of the larynx (Table 1 and Fig. 1).

**Table 1:** Aspiration prevention surgeries

Aspiration prevention surgeries		Types of anesthesia	Operative time	Amount of bleeding	Risk of suture failure	Possible postoperative speech	UES opening effect
**Surgeries to remove the larynx**	Total laryngectomy [14–19]	G	> 2 h	Relatively large	Relatively low	Eso-S/ VP	+
	Central-part laryngectomy [20–24]	G, L	≒ 2 h	Small	Low	Eso-S/ VP	+
**Surgeries to change the tracheal structure**	Tracheoesophageal diversion [4, 25–29]	G	> 2 h	Small	Relatively low	Eso-S/ VP	–
	Laryngotracheal separation [30–34]	G, L	≒ 2 h	Small	Low	–	–
	Tracheal flap method [35, 37, 38]	G, L	≒ 2 h	Small	Low	–	–
**Surgeries to close the larynx**	Supraglottic laryngeal closure						
	Epiglottic flap [1, 39, 40]	G	≒ 2 h	Small	Moderate	–	–
	Vertical laryngoplasty [41–43]	G	≒ 2 h	Small	Moderate	Possible in some cases	–
	Transoral supraglottic closure [44]	G	≒ 2 h	Small	Moderate	–	–
	Glottic laryngeal closure [21, 22, 24, 45–57]	G, L	≒ 2 h	Small	Low	–	with CPM*
	Subglottic laryngeal closure [21, 58, 59]	G, L	≒ 2 h	Small	Low	–	with CPM or TC*

*G* general anesthesia, *L* local anesthesia, *UES* upper esophageal sphincter, *≒ 2 h* around 2 h; *Eso-S* esophageal speech, *VP* voice prosthesis, *CPM* cricopharyngeal myotomy, *TC* total cricoidectomy

^*^Only in patients with cricopharyngeal myotomy or total cricoidectomy

### Surgeries to remove the larynx

#### Total laryngectomy

Total laryngectomy (Fig. 1B) is indicated for advanced laryngeal and pharyngeal cancers; it was also the treatment of choice for intractable aspiration before the 1970s [14]. It allows a definitive separation of the digestive and respiratory tracts. The larynx is exposed and skeletonized, and pharyngotomy and definitive laryngeal removal are performed [15–17]. Pharyngeal reconstruction is subsequently performed using direct closure, anterolateral thigh flap, free jejunal flap, or other techniques depending on the indication [18, 19]. Total laryngectomy is performed under general anesthesia; it is not commonly performed under local anesthesia. Total laryngectomy is a globally common procedure that can be performed by several head and neck surgeons. It is not recommended if there is a possibility of laryngeal function improvement since it is irreversible.

#### Central-part laryngectomy

Central-part laryngectomy (CPL) (Fig. 1C) is a type of narrow-field laryngectomy procedure first reported in Japan [20]. It is less invasive than total laryngectomy and involves the removal of the cricoid cartilage with the glottis, cutting the cricopharyngeal muscle, but preserving the epiglottis, the entire hypopharyngeal mucosa and major vessels and nerves running into the larynx [20]. CPL was proven to be effective in preventing aspiration pneumonia and may improve oral nutrition by reducing the resting upper esophageal sphincter (UES) pressure and prolonging UES relaxation during swallowing [21–24]. CPL is an effective procedure for patients with diseases such as amyotrophic lateral sclerosis, which is characterized by reduced general muscle strength or pharyngeal contraction during swallowing that makes passage of food boluses from the pharynx to the esophagus difficult.

### Surgeries to change the tracheal structure

#### Tracheoesophageal diversion

Lindeman in 1975 described a diverting procedure using a tracheoesophageal anastomosis for intractable aspiration (Fig. 1D) [25]. He initially performed the procedure on six mongrel dogs with successful outcomes. The procedure is usually performed under general anesthesia and involves dividing the trachea horizontally between the second and fourth tracheal rings. The proximal tracheal segment is anastomosed in an end-to-side fashion to an anterior esophagotomy. The distal tracheal segment is sutured to the anterior cervical skin, creating a tracheostomy. This technique has the following advantages: it allows secretions and orally ingested substances that have passed into the larynx to drain into the esophagus; tracheal reconstruction for reversal of the aspiration procedure is easier with the long proximal segment than with the short subglottic tracheal segment of the laryngotracheal separation (LTS) [4, 25, 26]. However, it is important to ensure that adequate tension-free anastomosis can be performed. If a high tracheostomy hole has already been created, which will prevent a tension-free anastomosis, LTS is preferred [4, 5, 27]. Although swallowing function did not improve after surgery, oral intake was possible, even for patients who presented with aspiration during pharyngeal swallowing and no swallowing reflex [17, 28, 29].

#### Laryngotracheal separation

Lindeman described a modified tracheoesophageal diversion procedure in 1976, the following year described tracheoesophageal diversion [30], which is now called laryngotracheal separation (Fig. 1E). LTS became popular globally and has been performed for several patients with intractable aspiration, including children and adults [30–34]. The procedure may be performed under local or general anesthesia. The trachea is divided between the second and third rings or at the level of the pre-existing tracheostomy, and the proximal trachea is closed anteroposteriorly over several layers. The distal tracheal segment is sutured to the inferior skin flap to create a wide tracheostomy. When a tracheostomy has been previously performed, LTS is recommended over tracheoesophageal diversion [4]. Avoiding tracheoesophageal anastomosis makes the procedure quicker and easier. No problems related to pooled material in the proximal pouch were encountered [4, 34]. There is also no risk of esophageal scarring, which can contribute to swallowing difficulties, since the esophagus is not breached. It is well accepted by patients and their families because the larynx is preserved [6].

#### Tracheal flap method

The tracheal closure or tracheal flap method was described by Ninomiya et al. in 2008 (Fig. 1F) [35]. A U-shaped flap of the tracheal anterior wall from the second to the fourth or fifth tracheal ring is created, bent toward the tracheal lumen, and sutured to the tracheal posterior/lateral walls by mattress stitches for tracheal closure. In addition, the closure was covered with a cutaneous U-shaped flap for reinforcement, and a permanent tracheal stoma was constructed. The main characteristic of this technique is the absence of tracheal transection. In tracheoesophageal diversion/LTS, the transected trachea is above the tracheal stoma. Therefore, a tracheal cannula may come into contact with the upper tracheal rings, which sometimes causes postoperative complications such as granulation and fistulation [36].

Shino et al. reported three subtypes of the tracheal flap method [37]: anterior tracheal flap, which is used for closure of the trachea for patients without previous tracheostomy (A-type); mucoperichondrial flap, which is used for patients who lack an anterior tracheal wall due to a previous tracheostomy (B-type); esophageal flap, which is used for patients with a habit of continuous suctioning of saliva due to hypersalivation (C-type). In all three subtypes, the tracheal closure site is covered by an anterior cervical skin flap [37, 38].

### Surgeries to close the larynx

#### Supraglottic laryngeal closure

##### Epiglottic flap

Habal and Murray reported epiglottic flap closure of the larynx in 1972 (Fig. 1G) [1]. The supraglottic larynx is closed by denuding the edges of the epiglottis, aryepiglottic folds, and arytenoids and suturing the epiglottis as a flap posteriorly to the aryepiglottic folds and arytenoids; a tracheostomy is required. Success with this technique occurred in approximately half of the procedures, but failures were successfully revised [5]. Strome and Fried added steps to prevent posterior flap dehiscence via the reduction of tension on the epiglottic flap by severing the hypoepiglottic and thyroepiglottic ligaments. Laurian et al. and Remacle et al. reported modified versions of the classical method in 1986 and 1998, respectively [39, 40]. The advantages of the epiglottic flap closure include reversibility, retention of swallowing, and speech preservation if the posterior laryngeal inlet is left open or dehiscence of the closure occurs. In addition, the true vocal cords are not injured with this procedure. The disadvantages of the epiglottic flap closure include a high rate of flap dehiscence and failure, the need for a transcervical approach with the possibility of superior laryngeal nerve injury, and supraglottic stenosis after reversal [5].

##### Vertical laryngoplasty

Biller et al. described a vertical laryngoplasty technique in 1983 for the prevention of aspiration in patients who required total glossectomy for advanced carcinoma of the tongue [41]. With this technique, incisions are made along the lateral margins of the epiglottic and aryepiglottic folds and over the arytenoids and interarytenoid area. The epiglottis and supraglottic larynx are closed vertically as a tube in two layers, with an opening at the top of the epiglottis. The resultant closure is chimney-shaped and consists of structures above the glottis. A small opening at the top of the tube allows for phonation, and the height of the tube protects the posterior space from airway contaminants. This technique has been applied to patients with intractable aspiration and has shown excellent results, allowing swallowing and speech in some patients. However, failure secondary to posterior dehiscence may limit the efficacy of this technique. To address the occasional leakage of the posterior part of the tubed laryngeal closure, Sato and Nakashima modified the technique of Biller et al. by first performing posterior glottis closure with the arytenoid and interarytenoid mucosal flaps, followed by supraglottic closure with the arytenoids and aryepiglottic folds [42]. Recently, attempts to perform this procedure using an endoscopic approach have also been reported [43].

##### Transoral supraglottic closure

Atallah and Castellanos described a surgical technique to eliminate aspiration that is performed entirely using reconstructive transoral laser microsurgery [44]. They created a barrier between the trachea and the pharynx using the epiglottic and aryepiglottic mucosa and the false vocal folds. A fully healed tracheostomy is required before the laryngeal procedure. After circumferentially incising and releasing the supraglottic mucosa, the distal tissues are sutured side-to-side longitudinally, and the superficial tissues are sutured back-to-front transversally. There is the risk of a small fistula with a leak; however, it can be repaired using a follow-up transoral procedure [44].

#### Glottic laryngeal closure (glottic closure)

Montgomery described the glottic closure procedure in 1975 (Fig. 1H) [45, 46]. During this procedure, the larynx is closed at the level of the true and false vocal cords. The larynx is opened via a midline thyrotomy, and the true and false vocal cords, ventricles, and the posterior commissure are denuded of the mucosa. Drawstring sutures are used to approximate the glottic surfaces for closure of the larynx, and a tracheostomy is necessary. Sasaki et al. modified the glottic closure procedure by adding a layer of laryngeal closure with a sternohyoid muscle flap [47]. Furthermore, advances in the prevention of postoperative stenosis of the tracheal stoma by combining partial removal of the cricopharyngeal cartilage with glottic closure [48, 49] and improvement of the passage of food through the upper esophageal portion by combining bilateral cricopharyngeal myotomy have been reported [22, 50]. The advantages of glottic laryngeal closure over total laryngectomy and tracheoesophageal diversion include a higher success rate for aspiration prevention, allowance of swallowing, and less invasiveness [49, 51]. Therefore, this procedure can be performed under local anesthesia for patients who are not good candidates for general anesthesia, such as patients with advanced head and neck cancers and neuromuscular diseases [21, 52–56]. In addition, this procedure was reported to improve the quality of life by preventing aspiration pneumonia, improving oral feeding status, and reducing suction frequency [24, 49, 57].

#### Subglottic laryngeal closure

Subglottic laryngeal closure may be the procedure of choice for the definitive separation of the upper alimentary and respiratory passages. During this procedure, the larynx is closed at the level of the subglottis (Fig. 1I). Eisele et al. described this procedure in 1995 [58]. They preserved the inner perichondrium of the cricoid cartilage, removed only the cricoid cartilage, and sutured the perichondrium at the subglottic level for the subglottic laryngeal closure. A permanent tracheostomy was created afterward. Later on, Miyake et al. [59] and Ueha et al. [21] modified the original method by leaving the posterior cricoid cartilage and adding cricopharyngeal myotomy. Subglottic laryngeal closure is possible under general or local anesthesia. Combined with a total cricothyrotomy or cricopharyngeal myotomy, the passage of the cervical esophagus is more likely to be improved [21].

### Others

#### Endolaryngeal stents

Several authors have reported the use of endolaryngeal stents to prevent aspiration [60–64]. These stents can be placed endoscopically, and the procedure is reversible with stent removal. A tracheostomy tube is necessary. The use of a vented silicone stent that permits phonation has been reported [64]. The disadvantages of using a stent to prevent aspiration include inconsistent outcomes due to leakage around the stent, the potential for endolaryngeal injury from the stent, and patient discomfort. Because of these drawbacks, the placement of endolaryngeal stents has failed to gain wide acceptance as a technique for the prevention of aspiration.

#### Vocal fold medialization

Vocal cord paralysis can result in chronic aspiration, particularly when combined with a laryngeal sensory deficit (e.g., a high vagal lesion) [65, 66]. This can be prevented with injection medialization laryngoplasty [67–72]. To medialize the vocal cords, vocal cord injection can be performed endoscopically, transcervically, or transorally. Temporary vocal cord medialization can be achieved using hemostatic compressed sponge (Gelfoam, Pfizer, New York, NY, USA) [73], collagen [74], or autologous fat [75] for injection. Meanwhile, calcium phosphate [69, 76] and hydroxyapatite [77] are not easily absorbed and are suitable for those who desire a permanent vocal fold medialization effect. The latter is more suitable for preventing aspiration.

Laryngeal framework surgery, which involves medialization thyroplasty and arytenoid adduction, is another excellent technique for vocal cord medialization [69, 78–80]. With medialization thyroplasty, Silastic (Dow Chemical, Midland, MI, USA) or another implant material is placed through a window in the thyroid cartilage to medialize the vocal cord. This procedure can be performed under local anesthesia, allowing tailoring of the implant shape and fine-tuning of implant placement while the patient phonates. Arytenoid adduction is also effective for medializing the paralyzed vocal cord, during which the arytenoid muscle is pulled across anteriorly by a suture until it is almost parallel to the lateral cricothyroid muscle. This is particularly helpful in closing posterior gaps between adducted vocal folds [81]. Postoperative swallowing studies have revealed the elimination of aspiration, enhanced pharyngeal clearance, and a capacity to liberalize bolus volume with consistency [78].

## Clinical outcomes and benefits

**Fig. 2:**
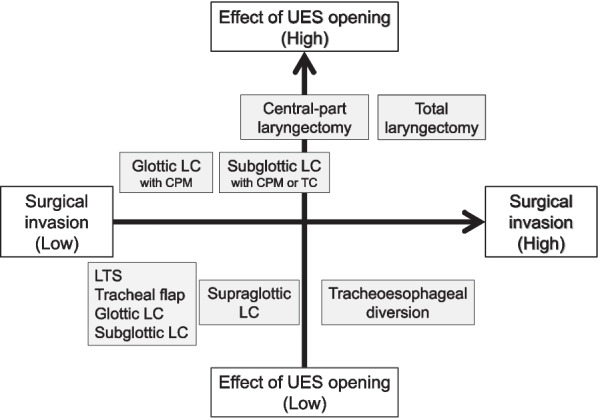
Classification of aspiration prevention surgeries based on surgical invasiveness and upper esophageal sphincter opening effects. *UES* upper esophageal sphincter, *LTS* laryngotracheal separation, *LC* laryngeal closure, CPM, cricopharyngeal myotomy, *TC* total cricoidectomy

APSs are performed to prevent aspiration pneumonia. Several reports have shown that APSs prevent aspiration and increase oral intake in 50%–80% of patients [24, 36, 49, 53, 82]. It is particularly effective for patients with higher levels of consciousness and mobility and those aged ≤ 30 years [7, 29]. The postoperative oral intake status can improve after an APS, but the influence of the disease background of the patients should be considered. The postoperative oral intake of patients with neuromuscular diseases, such as multiple system atrophy and amyotrophic lateral sclerosis as well as head and neck cancers with severe dysphagia has also been reported to improve after APS [52–55]. If the goal is to prevent aspiration and oral intake, the appropriate procedure should be selected from various APSs. For instance, surgical techniques, such as cricopharyngeal myotomy and total cricoidectomy, facilitate bolus passage of the UES section. They should be performed simultaneously with APS for patients with severe aspiration who require APS and have impaired UES opening or its risk, as aspiration prevention alone cannot sufficiently improve the UES passage of the food bolus (Fig. 2) [21, 22].

Complications after APS should be understood as well. Postoperative fistula or flap dehiscence caused by suture failure has been a major concern after APS. Postoperative suture failure is frequent after epiglottic flap closure and total laryngectomy. The postoperative success rates of epiglottic flap closure are within 50%–75%, with the most common complication being flap dehiscence of the posterior portion [5, 39]. For total laryngectomy, the most common postoperative complications and incidence reported were pharyngocutaneous fistula (28.5%), wound infection (28%), and pharyngeal stenosis (6%) [83, 84]. For tracheoesophageal diversion, only a few severe complications were reported, with postoperative fistulas being the major concern following this procedure, albeit it was infrequent [27, 28]. Suture failure and wound infection are rarely reported after CPL, LTS, glottic closure, and subglottic closure [24]. Although the duration of surgery and intraoperative bleeding after APSs are influenced by the skill of the surgeon and the anatomy of the patient, all surgeries, except total laryngectomy and tracheoesophageal diversion, can be completed within approximately 2 h with minimal blood loss [21]. Since some APSs can be performed under local anesthesia, a procedure should be chosen after considering the risk of complications and the general condition of the patients (Table 1) [21, 22].

An important benefit of APSs that should not be ignored is the improvement of the quality of life for patients and their caregivers [24, 49]. After APSs, the frequency of suctioning decreases in several cases. In addition to improving oral intake, a decrease in the suctioning frequency may contribute to an improvement in the quality of life of patients and caregivers. Moreover, by reducing the suction frequency and the risk of developing aspiration pneumonia, APSs may improve the quality of life of patients, especially those with limited life expectancy, as patients can opt to receive medical treatment at home. This is meaningful because being able to receive home care is of great importance to patients receiving palliative care [52].

## Speech after aspiration prevention surgery

Most patients lose vocal function after APSs. To restore oral communication, an electrolarynx is an option for alaryngeal speech [85]. A small hand-held vibrating device is placed against the neck or cheek. The vibration is introduced into the oral cavity and used as a sound source for speech. Esophageal speech is another technique of alaryngeal speech. Patients who have undergone laryngectomy for cancer produce speech sounds with airflow-induced vibrations of the pharyngoesophageal segment [86, 87]. Some patients with a tracheoesophageal puncture (TEP) after total laryngectomy can restore their speech function. TEP creates a path for air to move from the lungs to the esophagus, which results in a new tracheoesophageal voice [88–90]. TEP with voice prosthesis placement is the gold standard for voice restoration after total laryngectomy [91, 92]. TEP can be performed in patients with total laryngectomy as well as those who have undergone LTS or tracheoesophageal diversion [93–95]. In rare cases, patients who have undergone tracheoesophageal diversion may accidentally regain laryngeal speech without additional treatment or special training [96]. Regarding voice quality, the best sound quality is achieved by reacquisition of the voice through the vocal cords after APSs. Among various APS techniques, only tracheoesophageal diversion with tracheotracheal speech fistula [97] or TEP [36] can provide a definitive treatment of aspiration while maintaining the use of the vocal folds for phonation.

Given the disease background of patients undergoing APSs, the use of electrolarynx and esophageal speech with the TEP may not be appropriate compensatory speech methods. However, they may be considered for patients who may benefit from them.

## Choosing the appropriate aspiration prevention surgery

**Fig. 3:**
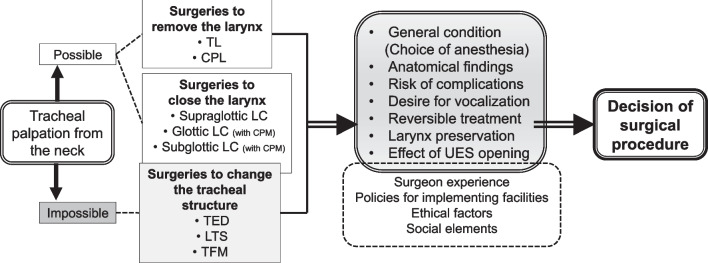
Selection process for aspiration prevention surgery procedure. *TL* total laryngectomy, *CPL* central-part laryngectomy, *LC* laryngeal closure, *CPM* cricopharyngeal myotomy, *TED* tracheoesophageal diversion, LTS, laryngotracheal separation, *TFM* tracheal flap method, *UES* upper esophageal sphincter

Surgeons’ experience and preferences as well as the policies and culture of the facility where the surgery is performed may influence the APS choice. In this section, we offer our personal opinions when selecting the appropriate APS for an individual patient (Fig. 3).

First, group the APSs that can and cannot be performed according to the patient’s age and anatomical characteristics. Determining whether the site of airway closure can be accessed from the neck is important. Compared with the larynx of children, elderly persons have a descending larynx, and the trachea is often not palpable from the neck. In patients with a descending larynx, performing APSs to change the tracheal structure, such as tracheoesophageal diversion, is difficult. If there is a neoplastic lesion in the neck, APSs should be performed after tumor resection or at a site where the airway lumen can be closed even in the presence of neoplastic lesion. Subsequently, as mentioned above, the surgical procedure should be selected based on the patient’s general condition, anatomical findings, complications (bleeding, suture failure, etc.), postoperative speech, reversibility, UES opening effect, and other characteristics of each surgical method (Fig. 3).

As an example, when performing APS in children with severe dysphagia who have potential for recovery with a long-term course, we will choose reversible tracheoesophageal diversion if general anesthesia is achievable. Meanwhile, for elderly patients in poor general condition who wants oral intake after surgery, we will choose glottic or subglottic closure, which can be performed under local anesthesia, in combination with cricopharyngeal myotomy. Hence, selecting an appropriate APSs according to the patients’ conditions is necessary.

## Conclusions

In this review, we described the history and development of APS. This review of APSs provides relevant insights that will guide clinicians in choosing the appropriate approach for patients who may benefit from them. Future APSs will require procedural innovations that will concurrently prevent aspiration and improve quality of life by improving oral intake, reducing suctioning frequency, and preserving speech function. Medical professionals need to continually endeavor to improve their knowledge and skills to facilitate the selection of appropriate APSs for the conditions of patients.

## Data Availability

Not applicable.

## Abbreviations

APS: Aspiration prevention surgery
CPL: Central-part laryngectomy
LTS: Laryngotracheal separation
TEP: Tracheoesophageal puncture
UES: Upper esophageal sphincter
