# Correction: Aspiration prevention surgeries: a review

**DOI:** 10.1186/s12931-023-02398-2

**Published:** 2023-05-03

**Authors:** Rumi Ueha, Redentor B. Magdayao, Misaki Koyama, Taku Sato, Takao Goto, Tatsuya Yamasoba

**Affiliations:** 1 Swallowing Center, The University of Tokyo Hospital, 7-3-1 Hongo, Bunkyo-Ku, Tokyo, 113-8655 Japan; 2 Department of Otolaryngology and Head and Neck Surgery, Faculty of Medicine, The University of Tokyo, Tokyo, Japan; 3 Department of Otorhinolaryngology-Head and Neck Surgery, Eastern Visayas Medical Center, Tacloban, Philippines

**Correction: Respiratory Research (2023) 24:43** 10.1186/s12931-023-02354-0

Following publication of the original article [1], the authors identified an error in Table 1 which occurred during the editing process of the publisher. The correct table (Table 1) is given in this correction.

**Table 1:** Aspiration prevention surgeries

Aspiration prevention surgeries		Types of anesthesia	Operative time	Amount of bleeding	Risk of suture failure	Possible postoperative speech	UES opening effect
**Surgeries to remove the larynx**	Total laryngectomy [14–19]	G	> 2 h	Relatively large	Relatively low	Eso-S/VP	+
	Central-part laryngectomy [20–24]	G, L	≒ 2 h	Small	Low	Eso-S/VP	+
**Surgeries to change the tracheal structure**	Tracheoesophageal diversion [4, 25–29]	G	> 2 h	Small	Relatively low	Eso-S/VP	−
	Laryngotracheal separation [30–34]	G, L	≒ 2 h	Small	Low	−	−
	Tracheal flap method [35, 37, 38]	G, L	≒ 2 h	Small	Low	−	−
**Surgeries to close the larynx**	Supraglottic laryngeal closure						
	Epiglottic flap [1, 39, 40]	G	≒ 2 h	Small	Moderate	−	−
	Vertical laryngoplasty [41–43]	G	≒ 2 h	Small	Moderate	Possible in some cases	−
	Transoral supraglottic closure [44]	G	≒ 2 h	Small	Moderate	−	−
	Glottic laryngeal closure [21, 22, 24, 45–57]	G, L	≒ 2 h	Small	Low	−	With CPM*
	Subglottic laryngeal closure [21, 58, 59]	G, L	≒ 2 h	Small	Low	−	With CPM or TC*

*G* general anesthesia, *L* local anesthesia, *UES* upper esophageal sphincter, *≒ 2 h* around 2 h; *Eso-S* esophageal speech, *VP* voice prosthesis, *CPM* cricopharyngeal myotomy, *TC* total cricoidectomy

*Only in patients with cricopharyngeal myotomy or total cricoidectomy

The original article has been updated.
